# Domestic violence against women during coronavirus (COVID-19) pandemic lockdown in Egypt: a cross-sectional study

**DOI:** 10.1186/s42506-022-00117-1

**Published:** 2022-11-14

**Authors:** Noha M. Abu Bakr Elsaid, Shaimaa A. Shehata, Haydy Hassan Sayed, Heba Saber Mohammed, Zeinab F. Abdel-Fatah

**Affiliations:** 1 Department of Public Health, Community, Environmental and Occupational Medicine, Faculty of Medicine, Suez Canal University, Fox Square, Ard El-gamayat, six building, flat 24, third district, Ismailia, 41511 Egypt; 2 Department of Basic Medical Sciences, Faculty of Medicine, King Salman International University, South Sinai, Egypt; 3 Department of Forensic Medicine and Clinical Toxicology, Faculty of Medicine, Suez Canal University, Ismailia, 41522 Egypt; 4 Department of Psychiatric and Neurological Diseases, Faculty of Medicine, Suez Canal University, Ismailia, Egypt; 5 Department of Obstetrics and Gynecology, Faculty of Medicine, Suez Canal University, Ismailia, Egypt

**Keywords:** Domestic violence, COVID-19, Prevalence, Risk factors, Women, Egypt

## Abstract

**Background:**

While it is necessary to limit the spread of the coronavirus (COVID-19) pandemic, efforts including social isolation, restricted travel, and school closures are anticipated to raise the probability of domestic violence (DV). This study aimed to estimate the prevalence, pattern, risk factors, and physical health outcomes of domestic violence against women during the COVID-19 pandemic.

**Methods:**

A cross-sectional study was conducted using a convenient sample. The data collection tool was based on Sect. 11 of the Egyptian Demographic Health Survey, 2014, which is designed to measure domestic violence. We used a Google form-designed questionnaire and distributed the link to social media platforms from May 2020 to June 2020 till the collection of the required sample of 388 completed questionnaires.

**Results:**

The prevalence of every form of DV was 31%. Emotional violence was the most prevalent (43.5%) followed by physical (38.9%) and sexual violence (17.5%). About 10.5% of women reported suffering from all types of violence. The husband was the most common perpetrator of DV. The determinants of ever experiencing any form of DV were low education level of women (OR = 7.3, 95% CI 2.8–18.8), unemployment (OR = 2.31, 95% CI 4–3.5), husband’s use of alcohol or substance (OR = 14.4, 95% CI 4.1–50.2), and insufficient income (OR = 2.01, 95% CI 2–3.2). The most common health consequences of DV were injuries such as cuts, bruises, and aches.

**Conclusions:**

The prevalence of ever experiencing any form of DV was 31% which is considered high. Emotional violence was the most common whereas sexual violence was the least common. Identifying the risk factors of DV would support the development and implementation of preventive and screening programs for early identification and offering social support to the victims. Policies should be adopted for the early detection and protection of women suffering from violent behaviors. Access to adequate prompt support and health-care services is crucial in order to decrease the consequences of violence. It is necessary to implement alcohol or drug abuse interventions, preventive measures, and screening programs in families to reduce DV.

## Introduction

As the novel coronavirus (COVID-19) pandemic has escalated globally, countries are adopting measures to reduce the spread of the virus [1]. Public health measures in the current pandemic such as quarantines, restricted travel, and channeling resources towards emergency service provision are likely to increase the risk of violence against women (VAW) [2]. Worldwide, VAW is considered as a critical public health problem [3]. During the COVID-19 pandemic, homes are no longer seen as safe zones; women and children are trapped with the perpetrators of violence [4]. Moreover, lack of access to regular social networks, social support, legal authorities, and other support services increased the burden of violence on women and children [3].

To lessen the spread of this pandemic, the World Health Organization (WHO) has recommended to stay-at-home. This leads to the rise in susceptibility to mental health problems due to experiencing a significant psychosocial stress [5, 6]. Socioeconomic conditions, education, substance use disorder or a mental disorder in the spouse, history of domestic violence (DV) during childhood, and family structure are known to increase violence risk [7]. Alcohol and substance use of the male was found to be highly associated with DV, and divorced or separated partners reported 2–3 times more violence than married ones [8].

Recently, several countries reported an alarming increase in DV cases linked to a pandemic such as China [9], the USA [10], Ethiopia [11], Canada [12], and Arab countries [13]. Although the increase in DV is temporary in line with the COVID-19 pandemic, its psychological negative consequences are expected to be long-lasting [14]. Exposure to violence causes disturbance in the mental health and life quality of women and increases the use of medical services [15].

The United Nations defined DV as “any act of gender-based violence that results in, or is likely to result in, physical, sexual, or mental harm or suffering to women” [16]. Domestic violence (DV) is a broad term and includes violence against parents, children, siblings, or even roommates and intimate partner violence (IPV) [17]. The WHO defined IPV as the “self-reported experience of one or more acts of physical and/or sexual violence by a current or former partner since the age of 15 years” [17]. Obviously, women who experience various forms of IPV will suffer from serious short- and long-term mental, physical, sexual, and reproductive health problems [18]. Previous studies have stated that neurological complications cerebral anoxia, bone fractures, burn, permanent infirmity, and even death were linked to IPV [19]. Domestic violence (DV) against women is a significant social and public health problem globally [20]. The WHO stated that about 1 in 3 women worldwide will encounter gender-based violence in their life while women in the Eastern Mediterranean Region have the third-highest prevalence of VAW worldwide [21]. Generally, DV during the pandemic could be attributed to many factors including economic stress, increased exposure to abusive relationships, disaster-related instability, and reduced access to support [22].

The United Nations Women Agency had provided guidelines to help governments to integrate gender perspectives into their response to a pandemic [23]. In addition, the WHO Health Emergency and Disaster Risk Management Framework recommended including gender-based violence services into the package of essential services provided during any crisis to minimize the risk of DV [24].

VAW appears to be a multi-causal complex phenomenon and has a great impact on the stability of family and children. DV is still an unidentified problem in Egypt and some females think that it is dangerous to report violence to their relatives, health care professionals, or medico-legal authorities [21]. Globally, VAW has been investigated during the COVID-19 pandemic; however, locally, there is still a lack of studies that explore its impact. This study aims to estimate the prevalence, describe the pattern, address the determinants, and describe the physical health consequences of DV against women during the COVID-19 pandemic in Egypt. The obtained data would help stakeholders to understand the current status and establish proper planning and implementation of national intervention programs to reduce women suffering.

## Methods

### Study design and setting

A cross-sectional analytic study was conducted. The study was conducted in Egypt which is the most populated country in the Arab world and the third most populous in the African continent, with about 103 million inhabitants as of 20,121. Egypt is divided into 27 governorates. Egypt governorates are geographically classified as follows: Upper Egypt, Lower Egypt, Urban governorates, and Frontier governorates.

### Sample size

The sample size was calculated using Epi info to be 352 at a 95% confidence interval (CI) level with a margin of error of ± 5% based on a previous reported prevalence of DV of 30% [15]. After adding a non-response rate of 10%, the total sample size was estimated to be 388 participants. However, we received 410 responses.

### Sampling technique

A convenient sample was used. Google form link was distributed via social media platforms in eight governorates till the collection of the required sample starting from May 2020 to June 2020. From each geographical zone, two governorates were selected. The eight governorates were red sea and new valley from the frontier governorates, Port Said, and Cairo from the urban governorates, Sohag and Minya from Upper Egypt, and Gharbia and Ismailia from Lower Egypt governorates.

### Inclusion criteria

Women aged 15 to 49 years lived in Egypt during the pandemic lockdown. Women who were educated (at least they can read and write to be able to fill the online questionnaire).

### Exclusion criteria

Women did not have Internet access so they could not access social media platforms. Women had residences outside the selected governorate.

### Data collection tools

An online self-administered Arabic questionnaire was used to collect data, which was adapted from Sect. 11 of the Egyptian Demographic and Health Survey (EDHS) 2014, which is devoted to estimating the prevalence of domestic violence [25]. EDHS is considered as a second nationally representative, cross-sectional, household survey that collects data from women of reproductive age (15–49 years).

The instrument is valid and reliable. The domestic violence module was previously tested and piloted in interviews conducted with eligible women in the subsample selected for the anemia-testing component of the EDHS [17]. Cronbach’s alpha for the emotional violence section was 0.79, for physical violence was 0.61, while for sexual violence, it was 0.59 and Cronbach’s alpha for the three sections was 0.67. The questionnaire measures three forms of violence emotional, physical, and sexual and consists of five sections. The first section included socio-demographic characteristics such as age, education, residence, and marital status. The second, third, and fourth sections consisted of questions regarding physical, sexual, and emotional violence, respectively. The response to these questions was (ever, often, sometimes, and often or sometimes). Physical violence exposure was evaluated by seven questions: “if perpetrator, husband, family member, or any other person” “(I) pushed, shacked, or threw something at her, (II) slapped her, (III) wrapped up her arm or pulled her hair, (IV) punched her with his fist or something that could hurt, (V) kicked or dragged her, (VI) attempted to choke or burn her, (VII) threatened or assailant her with a knife, gun or other firearms.” Sexual violence exposure was assessed by answering three questions: (I) if the perpetrator had physically forced her to have sexual intercourse with him when she did not want to, (II) physically forced her to perform any other sexual acts she did not want to, and (III) forced her with threats or in any other way to perform sexual acts she did not want to. Emotional violence was assessed by, “(I) Said or did something to demean her in front of others, (II) threatened to hurt or injury her or the person she looked after, and (III) made her feel bad about herself.” In the current study, the responses of the studied group who had at least one positive response to any form of violence were acceptable as exposure to DV. In the fifth section, women were asked questions about the perpetrator of violence, exposure to violence during pregnancy, types of injuries caused by DV (e.g., soft tissue injuries, deep wounds, burns, bones fractures, and broken teeth), and any trials at help-seeking for domestic violence during the COVID-19 pandemic.

### Statistical analysis

The data were analyzed using Statistical Package for Social Sciences (SPSS) version 23 IBM Corp. Released 2015. IBM SPSS Statistics for Windows, Version 23.0. Armonk, NY: IBM Corp. Continuous variables were presented as mean and standard deviation, while categorical variables were presented as frequency and percentage. For inferential statistics, Pearson’s chi-square was used to test the relation between DV and the qualitative independent variables. A *p* value ≤ 0.05 was considered statistically significant. A logistic binary regression model was used to predict the odds of being violent. *p* ≤ 0.05 was considered statistically significant with 95% CI.

### Ethical considerations

The study was approved by the Research Ethics Committee of Suez Canal University with approval number 4177. Informed consent was written at the beginning of the Google form. The participants were informed that participation in the study is voluntary and that they can refuse to respond without stating any reason. The collected data were anonymous and confidential and will be used only for research.

## Results

Among the 410 women, 43.4% of the participants were in the age groups 25–34 years, 87.6% lived in an urban area, 47.8% were highly educated, 65% were employed, and nearly 80% of participants reported their monthly income as sufficient. Regarding marital status, 79.8% were married with a mean duration of marriage of 8.8 ± 7.1 years, a small proportion of participants (8.2%) were pregnant, and 47.6% of the participants had from one to two children. As regards husband education, nearly 70% of them (69.8%) had secondary or higher education degree (Table 1).

**Table 1:** Socio-demographic characteristics of the studied women in eight governorates, Egypt, 2020 (*n* = 410)

**Variable**	**Frequency**	**Percent (%)**
**Age**		
15–24	57	13.9
25–34	178	43.4
35–49	175	42.7
**Number of living children**		
0	92	22.4
< 3	195	47.6
≥ 3	123	30
**Marital status**		
Married	327	79.8
Single	62	15.1
Divorced	18	4.4
Widowed	3	0.7
**Pregnant women (*n* = 327)**	27	8.2
**Residence**		
Urban	359	87.6
Rural	51	12.4
**Education**		
Primary education	29	7.1
Secondary education/higher	185	45.1
Postgraduate	196	47.8
**Husband’s education (*n* = 348)**		
Primary complete/some secondary	6	1.7
Secondary complete/higher	243	69.8
Postgraduate	99	28.5
**Work status**		
Working for cash	265	64.6
Not working for cash	145	35.4
**Age at marriage (*n* = 348)**		
**< 20 years**	46	13.2
**20–30 years**	293	84.2
**≥ 30 years**	9	2.6
**Mean ± SD**	20.14	9.1
**Duration of marriage (*n* = 348)**		
**< 5 years**	71	20.4
**5–10 years**	126	36.2
**≥ 10 years**	151	43.4
**Mean ± SD**	8.8	7. 1
**Health problem**		
**Yes**	122	29.8
**No**	288	70.2
**Monthly income**		
**Sufficient**	325	79.3
**Not sufficient**	85	20.7
**Total**	410	100

**Fig. 1:**
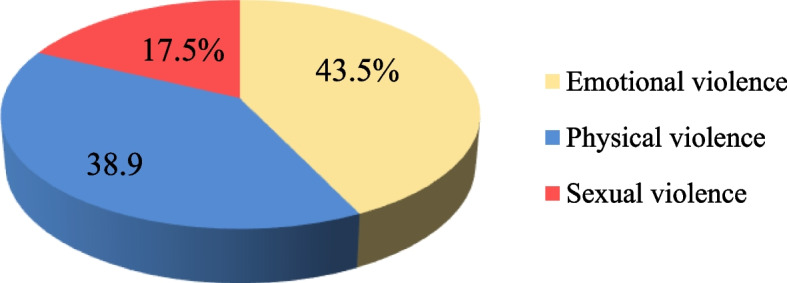
Prevalence of types of domestic violence among the studied women in eight governorates, Egypt, 2020 (*n* = 410)

As shown in Table 2 and Fig. 1, 31% reported exposure to violence. The highest percentage of participants (43.5%) was exposed to emotional violence and 39% to physical whereas 17% were abused by sexual violence.

**Table 2:** The prevalence of domestic violence among study participants, Egypt, 2020 (*n* = 410)

**The prevalence of violence**	**Violent**		**Not violent**	
	**No.**	**%**	**No.**	**%**
Ever violent (*n* = 410)	127	31	283	69
All types of violence (*n* = 410)	43	10.5	367	89.5

**Fig. 2:**
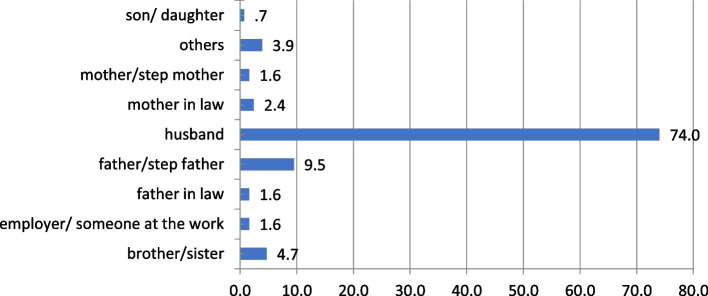
Percentage of the persons who committed the violence against study participants (*n* = 127)

**Fig. 3:**
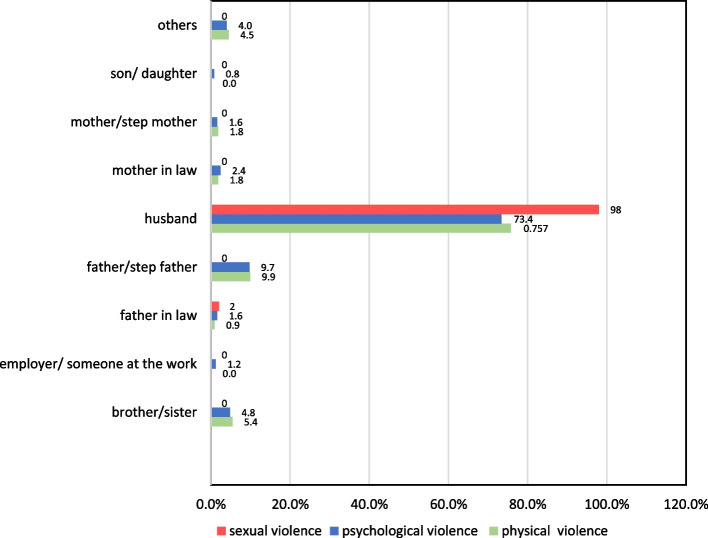
Percentage of the persons who committed the violence in each type of domestic violence

The husband was the most common perpetrator of every form of DV (74.02%) and in all forms of violence as shown in Figs. 2 and 3.

Table 3 showed the relationship between any form of violence and the socio-demographic characteristics of participants. There was a statistically significant difference between violent and nonviolent groups regarding marital status, women’s education, husband’s education, husband’s addictions, working status, age at marriage, and monthly income. Being divorced or widowed, had a primary level of education, low husband’s education level, husband’s addictions, not working, and early age at marriage increase the risk of any form of violence.

**Table 3:** The relationship between the demographic characteristics and domestic violence among the studied women in eight governorates, Egypt, 2020 (*n* = 410)

**Variable**	**No**	**Violent** ***N* (%)**	**Not violent** ***N* (%)**	***P* value** **Chi-square**
**Age**				
15–24	57	19 (33.3)	38 (66.7)	0.417
25–34	178	49 (27.5)	129 (72.5)	
35–49	175	59 (33.7)	116 (66.3)	
**Number of living children**				
0	92	28 (30.4)	64 (69.6)	0.763
< 3	195	63 (32.3)	132 (67.7)	
≥ 3	123	36 (29.3)	87 (70.7)	
**Marital status**				
Married	327	95 (29.0)	232 (70.9)	0.000*∆
Single	62	18 (29)	44 (71)	
Divorced	18	11 (61.1)	7 (38.9)	
Widow	3	3 (100)	0	
**Pregnant women**	27	25 (92.6)	2 (7.4)	
**Residence**				
Urban	359	109 (30.4)	250 (69.6)	0.287
Rural	51	18 (35.3)	33 (64.7)	
**Women’s education**				
Primary education	29	22 (75.9)	7 (24.1)	0.001*∆
Secondary education/higher	185	58 (31.4)	127 (68.6)	
Postgraduate	196	47 (24)	149 (76)	
**Husband’s education (*n* = 109)**				
Primary complete/some secondary	6	5 (83.3)	1 (16.7)	0.000*∆
Secondary complete/higher	243	86 (35.4)	157 (64.6)	
Postgraduate	99	18 (18.2)	81 (81.8)	
**Husband adduction**				
Yes	20	17 (85)	3 (15)	0.000*
No	390	110 (28.2)	280 (71.8)	
**Work status**				
Working for cash	265	65 (24.5)	200 (75.5)	0.000*
Not working for cash	145	62 (42.8)	83 (57.2)	
**Age at marriage (*n* = 109)**				
< 20 years	46	25 (54.3)	21 (45.7)	0.002*∆
20–30 years	293	81 (27.6)	212 (72.4)	
≥ 30 years	9	3 (33.3)	6 (66.7)	
**Duration of marriage (*n* = 109)**				
< 5 years	71	23 (32.4)	48 (67.6)	0.276
5–10 years	126	33 (26.2)	93 (73.8)	
≥ 10 years	151	53 (35.1)	98 (64.9)	
**Monthly income**				
Sufficient	325	90 (27.7)	235 (72.3)	0.005*
Not sufficient	85	37 (43.6)	48 (56.4)	

**∆** Fisher’s exact test

* Significant

Table 4 shows the comparison of the socio-demographic characteristics of participants with the different types of violence. The age group 35–49 years, married, primary education, husbands’ primary education, husbands’ addiction, not working, and early age at marriage had the highest prevalence of sexual violence. Being divorced or widowed, had a primary level of education, low husband’s education level, husband’s addictions, not working, low monthly income, and early age at marriage increase the risk of physical and emotional violence.

**Table 4:** Relationship between the socio-demographic characteristics and women’s exposure to different forms of violence, Egypt, 2020

**Variable**	**Physical violence** ***N* (%)** ***N* = 111**	***P* value**	**Emotional violence** ***N* (%)** ***N* = 124**	***P* value**	**Sexual violence** ***N* (%)** ***N* = 50**	***P* value**
**Age**						
15–24	18 (31.6)	0.46	18 (31.6)	0.57	3 (5.3)	**0.001**
25–34	43 (24.2)		49 (27.5)		11 (6.2)	
35–49	50 (28.6)		57 (32.6)		36 (20.6)	
**Marital status**						
Married	82 (25.1)	0.01*	92 (28.1)	0.002*	50 (15.3)	**0.002∆**
Single	17 (27.4)		18 (29)		0	
Divorced	10 (55.6)		11 (61.1)		0	
Widow	2 (66.7)		3 (100)		0	
**Women’s education**						
Primary education	18 (62.1)	0.001*	21 (72.4)	0.001*	8 (27.6)	0.007*∆
Secondary education/higher	51 (27.6)		56 (30.3)		26 (14.1)	
Postgraduate	42 (21.4)		47 (24)		16 (8.2)	
**Husband’s education**						
Primary education	5 (83.3)	0.000*∆	5 (83.3)	0.000*	2 (33.3)	0.000*∆
Secondary education /higher	76 (31.3)		85 (35)		42 (17.3)	
Postgraduate	13 (13.1)		16 (16.2)		6 (6.1)	
**Husband addiction**						
Yes	17 (85)	0.000*	17 (85)	0.000*	11 (55)	0.000*
No	94 (24.1)		107 (27.4)		39 (10)	
**Work status**						
Working for cash	58 (21.9)	0.001*	63 (23.8)	0.000*	19 (7.2)	0.000*
Not working for cash	53 (36.6)		61 (42.1)		31 (21.4)	
**Monthly income**						
Sufficient	76 (23.4)	0.001*	87 (26.8)	0.003*	39 (12)	0.813
Not sufficient	35 (41.2)		37 (43.5)		11 (12.9)	
**Age at marriage**						
< 20 years	24 (52.2)	0.000*∆	25 (54.3)	0.003*∆	16 (34.8)	0.000*∆
20–30 years	68 (23.2)		78 (26.6)		33 (11.3)	
≥ 30 years	2 (22.2)		3 (33.3)		1 (11.1)	
**Total**	94		106		50	

**∆** Fisher’s exact test

* Significant

**Fig. 4:**
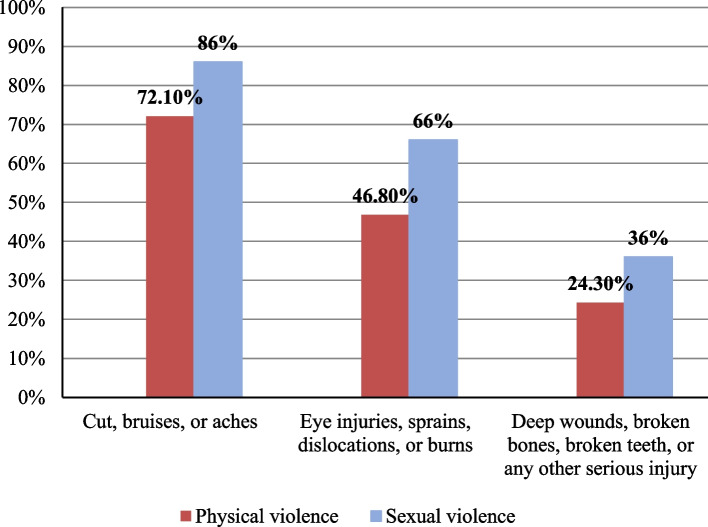
Percentage of injuries resulting from different forms of violence among the studied women in eight governorates, Egypt, 2020

Figure 4 exhibited different forms of traumatic injuries among battered women. Interestingly, women who experienced sexual violence were more injured than women who only experienced physical violence.

Table 5 showed a logistic regression analysis of predictors of every form of violence and each form of violence. Significant predictors associated with exposure to every form of DV were the following: primarily educated women (OR = 7.3), unemployment (OR = 2.3), husband’s use of alcohol or drugs (OR = 14.4), and insufficient income (OR = 2.0). The significant predictors of physical violence were primarily educated women (OR = 4.5), unemployment (OR = 2.06), husband’s use of alcohol or drugs (OR = 17.8), and insufficient income (OR = 2.2). The most common predictors of emotional violence were primary educated women (OR = 6.5) and husbands’ addiction (OR = 14.9), whereas the predictors of sexual violence were women aged 35 to 49 years old (OR = 4.6) and husbands’ addiction (OR = 14).

**Table 5:** Binary logistic regression analysis of factors associated with each type of domestic violence

**Variable**	**Physical violence**		**Emotional violence**		**Sexual violence**		**Total violence**	
	**OR**	**95% CI**	**OR**	**95% CI**	**OR**	**95% CI**	**OR**	**95% CI**
**Age** (15–24)^a^								
25–34	0.6	0.3–1.3	0.82	0.4–1.5	1.1	0.3–4.4	0.7	0.4–1.4
35–49	0.8	0.4–1.6	1.04	0.5–1.9	4.6	1.3–15.7	1.01	0.5–1.9
**Marital status** (married)^a^ **Unmarried**	1.6	0.9–2.6	1.6	0.9–2.6	-	-	1.5	0.9–2.5
**Women’s education** (secondary education/higher)^a^								
Primary education	4.5	1.9–10.7	6.5	2.6–16.4	2.2	0.8–5.8	7.3	2.8–18.8
Postgraduate	0.6	0.4–1.1	0.7	0.4–1.1	0.6	0.3–1.1	0.6	0.4–1.1
**Husband’s education** (secondary education /higher)^a^								
Primary education	9.6	1.0–18.5	7.8	0.8–17.2	1.9	0.3–12.2	7.8	0.8–20.5
Postgraduate	0.3	0.1–0.6	0.3	0.1–0.6	0.3	0.1–0.7	0.3	0.1–0.6
**Husband addiction** Yes	17.8	5.1–62.2	14.9	4.3–52.1	11	4.2–28.1	14.4	4.1–50.2
**Monthly income (sufficient)**^a^ Not sufficient	2.2	1.3–3.7	2.1	1.2–3.4	1.09	0.5–2.2	2.01	1.2–3.2
**Work status** (Working for cash)^a^ Not working for cash	2.06	1.3–3.2	2.33	1.5–3.6	3.52	1.9–6.5	2.3	1.4–3.5
**Age at marriage (≥ **30 years)^a^								
< 20 years	3.8	0.7–20.3	2.3	0.5–10.7	4.2	0.4–37.2	2.3	0.5–10.0
20–30 years	1.0	0.2–5.2	0.7	0.1–2.9	1.0	0.1–8.3	0.7	0.1–3.1

*OR* Odds ratio, *CI* Confidence interval

^a^Reference group

## Discussion

Domestic violence is a long-standing public health issue in the Arab world that is predicted to worsen in the aftermath of the COVID-19 epidemic [26, 27]. This is an alarming situation which needs immediate attention because according to Rabbani et al., the psychosocial results of domestic violence are serious and include the use of drugs, alcohol consumption, depression, and suicidal attempts [28].

Our study found that the prevalence of every form of violence during the COVID-19 pandemic was 31%, with the emotional violence as the most common (43.5%), followed by physical (38.9%), and sexual violence (17.5%). A small proportion of women suffered from all forms of violence. Our findings were relatively low when compared to another study conducted among married women in the Arab countries during the COVID19 pandemic which reported that half of the participants had been exposed to every form of violence. However, the ranking was like us; emotional violence was the most commonly reported (30.6%), followed by physical (14.3%), and sexual violence (13.5%), respectively [13]. The disparity in estimated prevalence could be explained by the diversity of methods, variation in DV definition, the fact that this issue is extremely sensitive in Egypt, and the fact that online users have higher socioeconomic levels than the general population.

Although the prevalence of DV in this study appears to be nearly similar to the rate previously reported by EDHS, 2014, in Egypt (30%) among ever-married women aged 15–49, the true prevalence of DV is underestimated during the pandemic due to several reasons. There is a change in the pattern of each type of violence during the pandemic compared with before where the most commonly reported form was 25% physical, 19% emotional, and 4% sexual in 2014 [25]. Our explanation for the underestimation of true DV prevalence during the pandemic is that the majority of the women in our sample were from a high socioeconomic class, were highly educated, lived in urban regions, and had active social media accounts.

On contrary, our findings were higher and had a different pattern than previously reported in Egypt by Habib et al. [29] who found that the most common form was physical abuse (29.9%), followed by sexual (7.8%), and emotional (6.6%). Our explanation is that during COVID-19, there was no solution to escape from domestic violence as most people have to stay at home most of the day, making violence towards women stronger and taking different forms, including threats, verbal, and physical abuse. Besides, during the COVID-19 pandemic, the home was turned into a dangerous place for partner violence victims because they had to spend prolonged hours with their partners and detached from people who support them [14].

We found that the most common perpetrator of every DV or each type was the husband. Working from home increased the levels of stress and anxiety among many family members. Isolation and confinement may trigger tensions leading to domestic violence. As a result, the perpetrators of abuse extended their power. The disturbances that accompanied the pandemic also limited access to services. Moreover, the need to stay at home in order to avoid COVID-19 together with the weak socioeconomic status of many women negatively impacted women and children who are most prone to domestic violence.

Also, the current study found that most of the pregnant women had been exposed to DV. This is much higher than previously reported in Egypt (only Seven percent) [25], seven facility-based studies (range from 10.4 to 34.6%) [26], DHS surveys in Comoros (three percent) [30] (34), and in Jordan (seven percent) [31]. These findings were consistent with worldwide Reports from China [9], France [32], Bangladesh [33], the USA [10], and Iran [34], where DV increased since they initiated a March lockdown.

Concerning risk factors associated with every form of DV, our study showed that a low level of education (some primary), not working, early age at marriage (< 20 years), husband’s abuse of alcohol or drugs, insufficient income were associated with her exposure to any form of DV. These findings were consistent with other studies [35–40]. All forms of violence were significantly higher in the low level of education of women and unemployed women. These findings indicated the importance of education to lower DV. The association between not working and violence might be explained that they might feel dependent on their partners so they accept and tolerate the violence.

Our study found that alcohol or drug abuse was the most common predictor of every form of DV or each type of violence. Drug addiction is a serious issue that worsens domestic violence. A recent study of 938 women in the city of Vitória, Espírito Santo, Brazil, found that alcohol and drugs user were more vulnerable to domestic violence [41]. Stress from the COVID-19 pandemic is a risk factor for alcohol and drug use/abuse to decrease negative feelings such as lack of control, financial worries, and fear of death [42]. Due to reduced supply during quarantine, anxiety, depressive symptoms, and withdrawal syndrome can be aggravated in alcohol and drug users. These symptoms may lead to more aggressive behavior in individuals with dysfunctional personality traits or personality disorders [43]. Impulsivity could lead to increased substance consumption or relapse and intensify tendencies toward domestic violence [44].

No association was found between violence and other socio-demographic variables like residence and number of siblings. These findings are consistent with previous studies [39–48] and contrary to findings previously reported by the EDHS that found violence tends to increase with the number of siblings and women living in rural Upper Egypt [25].

Cuts and bruises were the most common injuries rather than deep wounds or serious injuries. Interestingly, physical injuries were associated with sexual violence more than physical violence. These observations were consistent with previous findings by the EDHS [25]. However, there was an increase in the percentage of women who experience injuries during the pandemic. Several studies reported that the incidence and severity of physical violence during the pandemic were high compared with previous years [49–51]. This high rate could be explained by the delay of abused women reaching health care services until the late stages of the abuse cycle [52, 53]. The proportion of women who complained of cuts and bruises due to sexual violence and physical violence were 86% and 72.1% compared with 59% and 37%, respectively, in 2014 [25]. The proportion of women who complained of deep wounds, broken bones or teeth, or other serious injuries due to sexual and physical violence was 36% and 24.3%, respectively, compared with 18% and 7%, respectively, in Egypt [25].

Considering help-seeking behavior, our results show that most of the victims seek help compared to only one third of women in Egypt [21], contrary to Wali et al. who found that 97.2% of victims were reluctant to seek help [54]. This may be explained by the fact that nearly half of the participants in our study are postgraduates and have good awareness regarding violence.

### Strengths and limitations

To our knowledge, this study is one of the earliest studies to discuss violence in Egypt during the COVID-19 pandemic lockdown. Limitations of the study include using a cross-sectional study design which limits the ability to draw a causal inference. Although we selected our sample from a total of eight governorates trying to represent four regions of Egypt, still our sample was a connivant one so we cannot generalize our results. Additionally, the study includes women who only have access to the internet which may have led to limited access to different socioeconomic categories in the community and underestimate the true prevalence of DV in Egypt.

## Conclusions

The prevalence of every form of DV was 31%, which is considered high. Emotional violence was the most common whereas sexual violence was the least common. The husband is the most common perpetrator of DV. The low level of education, not working, husband’s use of alcohol or drugs, and insufficient income were potential risk factors for every form of DV. Identifying the risk factors of DV would support the development and implementation of preventive and screening programs for early identification and offering social support to the victims. Policies should be adopted for the early detection and protection of women suffering from violent behavior. Access to adequate, prompt support, and healthcare services in order to decrease the consequences of violence and provide an appropriate response considering the family context is essential. Psychological interventions and mental health services for people who already have mental disorders or who have developed them at the time of COVID-19 should be provided. The present study highlights the significant role of alcohol or drug abuse as a risk factor for domestic violence that indicates the necessity to implement preventive measures and screening programs in families and abuse intervention policies.

## Data Availability

The datasets used and analyzed during the current study are available from the corresponding author upon reasonable request.

## Abbreviations

DV: Domestic violence
EDHS: Egyptian Demographic and Health Survey
IPV: Intimate partner violence
SPSS: Statistical Package for Social Sciences
VAW: Violence against women
WHO: World Health Organization
